# The relationship between personality traits and willingness to undergo cosmetic surgery in the non-clinical population – a systematic review and meta-analysis

**DOI:** 10.3389/fpsyg.2023.1241952

**Published:** 2023-09-08

**Authors:** Gianina-Mălina Lăzărescu, Mona Vintilă

**Affiliations:** Faculty of Sociology and Psychology, West University of Timisoara, Timisoara, Romania

**Keywords:** cosmetic surgery, personality traits, meta-analysis, systematic review, perfectionism, appearance-based rejection sensitivity

## Abstract

**Objective:**

Conducting a systematic review and meta-analysis to synthesize previously obtained results regarding the relationship between interest in cosmetic surgery and personality traits.

**Methodology:**

A series of criteria were applied (at the level of design, independent variables, dependent variable, participants) in order to decide which existing studies could be considered eligible for inclusion in the meta-analytic procedure. The identification of research that met the eligibility criteria was carried out with the help of the electronic search function in the following databases: ScienceDirect, PsycInfo, Web of Science, Scopus, Springer, and PubMed. Following this approach left 13 studies that were then subjected to the final analysis and included in the meta-analysis.

**Results:**

The researchers’ expectations were partially supported by the results of the analyses, thus demonstrating the existence of a significant relationship between perfectionism (socially prescribed perfectionism; perfectionistic self-promotion), appearance-based rejection sensitivity, and interest in pursuing esthetic surgery.

**Discussions:**

Identifying these relationships will allow cosmetic surgeons to understand both the mechanisms underlying this decision and the need for psychological assessment/counseling before patients undergo such procedures. It will also allow psychologists to develop best practice guidelines for how they relate to the patient before they perform cosmetic surgery. At the same time, psychotherapists will be able to devise targeted and personalized interventions for each personality profile, so that the decision to undergo an esthetic operation is not made based on a dispositional trait (fear of rejection, stress caused by body dissatisfaction).

## Introduction

In recent times, we have been witnessing a considerable increase in the number of people wishing to modify certain parts of their bodies by means of various techniques and esthetic interventions. According to surveys carried out by the International Society of Aesthetic Plastic Surgery, from 2020 to 2021 there was a 19.3% increase in esthetic procedures performed globally, with liposuction being the most common surgical procedure and Botox injection the most common non-surgical one. We can also observe that, in 2021 as in 2020, breast augmentation was the most common surgical procedure among women and blepharoplasty among men. Turning to non-surgical interventions, treatment with Botox was the most popular among both women and men, with a striking interest in this procedure among young people of 18 or even less; 106,033 such procedures were performed on young people aged 18 or under in 2021 (International Society of Aesthetic Plastic Surgery, 2021).

These statistics illustrate the desire felt by very many people to improve their physical appearance in order to feel better about their own bodies, even from a very tender age; this phenomenon can be seen in all age categories (>18 to over 65). Although this desire is understandable, the decision to undergo cosmetic surgery needs to be taken after due consideration of all the potential effects (both positive and negative) of an intervention of this type, since minimizing the risks and not giving detailed attention to them can lead later to results that affect patients’ physical and psychological well-being (Gilmartin, 2011; Lorenzo et al., 2018; Lăzărescu and Vintilă, 2023). Besides the importance of patients being informed before they take such a major decision, cosmetic surgeons too should discover the motivation of anyone who wants to undergo such an intervention and evaluate it in order to identify possible disorders that could contribute to a low quality of life for the patient after the performance of the procedure (Vindigni et al., 2002; Qureshi et al., 2016). Thus, it is essential for doctors to identify the reasons underlying the desire to undergo cosmetic surgery so as to be able to offer services that help improve the quality of life (physical and mental) of patients.

Cosmetic surgery is a form of biomedical practice performed through various types of invasive procedures that aim to modify the patient’s bodily features so that he perceives his physical appearance as more desirable and attractive (Dean et al., 2018). Studies have shown that the main motivations of people who want to undergo an esthetic operation are social (messages received from parents, friends and partners, and also those circulating in social media) and intrapersonal (negative self-image) (Haas et al., 2008; Callaghan et al., 2011). In addition, people resort to cosmetic surgery out of a desire to redefine aging in a positive way, believing that they can defy this natural process by following cosmetic procedures (Bayer, 2005) or by purchasing certain products to make them look younger (Bytheway, apud Faircloth, 2003). Regarding adults, Slevec and Tiggemann (2010) state that women between the ages of 35 and 55 turn to cosmetic surgery due to body dissatisfaction, aging anxiety, exposure to media messages and the desire to invest in appearance. If we consider teenagers, the study by Lunde (2013) states that young men tend to resort to cosmetic surgery for social reasons (their attitudes are closely related to what those around them think about their physical appearance), while young women’s strongest motivation is the internalization of the ideal of physical beauty promoted by society; using filters before posting pictures on social networks is also an important predictor for the desire to undergo an esthetic operation (Maes and de Lenne, 2022). Given the increasing willingness of people to make use of cosmetic surgery, there is a need for practitioners to have at their disposal validated instruments that allow the assessment of patients’ attitudes toward such procedures (Sarwer et al., 2005). The most frequently used instrument in this regard is the 15-item Acceptance of Cosmetic Surgery Scale, which measures attitudes toward cosmetic surgery in terms of three subscales: Social (the decision to undergo such a procedure is based on social reasons), Intrapersonal (personal benefits of cosmetic surgery) and Consider (likelihood of undergoing cosmetic surgery in the future) (Henderson-King and Henderson-King, 2005). Its psychometric qualities have been proven through studies conducted in various countries (Brazil – Swami et al., 2011; Italy – Stefanile et al., 2014; Romania – Lazarescu et al., 2023; Turkey – Karaca et al., 2017; Hungary – Meskó and Láng, 2021, etc.).

As can be seen, the reasons underlying interest in following an esthetic intervention are quite varied for all age categories and we already have a well-known instrument to assess people’s motivation for embarking on such a procedure, which is precisely why it is essential to develop theoretical models that present, at a general level and for the entire population, social and intrapersonal predictors of the desire to undergo an esthetic operation.

### The tripartite influence model and body objectification theory

Over time, several theoretical models have been tried in an attempt to explain the psychological mechanisms behind the decision to undergo an esthetic procedure. Most previous research studies have as their theoretical basis the tripartite influence model (Thompson et al., 1999), which highlights the fact that there are three main sources that transmit, influence and reinforce cultural ideas of beauty: mass media, parents and colleagues. The mechanism behind these social reasons, which are the basis of the desire to have esthetic plastic surgery, involves the person in question internalizing messages from their immediate environment regarding their physical appearance (Jefferson and Stake, 2009; Haboush et al., 2012). Following the internalization of these messages, the person may come to compare his own body with those of other people who conform to the standard of beauty established by society (Festinger, 1954), an action that may lead to negative feelings toward his own person (Wheeler and Miyake, 1992) and the desire to modify certain body parts (Wu et al., 2022a,b). Although the model was originally developed to explain body image concerns and eating disorders, in recent years research has expanded its applicability to include people’s tanning behavior (Cafri et al., 2009) and attitudes toward cosmetic surgery in general (Menzel et al., 2011).

Based on the tripartite influence model, we can observe how external factors, such as social media or interactions with those close to you (friends, colleagues, family), contribute to the internalization of the culturally and societally imposed model of beauty, to the comparing of your physical appearance with that of others, and subsequently to the desire to undergo cosmetic surgery to change certain parts of the body with which you are not satisfied.

Another perspective that attempts to explain the motivation behind interest in pursuing cosmetic surgery is body objectification theory (Fredrickson and Roberts, 1997), which focuses on how social and cultural representations contribute to the process by which people internalize the observer’s perspective on their own body and come to believe that their personal value is given only by their physical appearance. When people attach particular importance to physical appearance and engage in social comparison with other people whose type of beauty falls within societally imposed standards (Lindner et al., 2012), they can feel body shame (Tiggemann and Boundy, 2008; Choma et al., 2009; Manago et al., 2015), body dissatisfaction (Grippo and Hill, 2008; Krawczyk and Thompson, 2015; Perez et al., 2018) and low self-esteem (Strelan et al., 2003; Adams et al., 2017; Breslow et al., 2020). When this phenomenon occurs, people may want to modify certain aspects of their body by resorting to various types of strategies: pursuing an esthetic operation (Ching and Xu, 2019; Skowronski et al., 2022; Schettino et al., 2023), for example, or following restrictive diets (Dakanalis et al., 2015; Schaefer et al., 2018) which over time can lead to the development of eating disorders (Mitchell and Mazzeo, 2009; Jongenelis et al., 2014). Another strategy is compulsive attendance at a gym to prevent weight gain (Grieve and Helmick, 2008; Prichard and Tiggemann, 2008; Du et al., 2023). It can thus be observed how the tendency of people to live on the basis of physical appearance objectification can lead to a series of negative phenomena that reflect a determination to change the body.

It is vital to mention that this theory can be associated in different situations with the tripartite influence model, which proposed three sources of influence (media, parents and peers) that contribute to people’s attitude toward strategies to change their physical appearance. Internalizing messages from these three sources of influence can lead to self-objectification of the body, which over time can lead to a desire for change.

### Personality and the desire to undergo cosmetic surgery

In addition to the two major perspectives that have tried to explain the motivation underlying the decision to request an esthetic surgical procedure, the specialist literature also regards personality as a determining factor for interest in such operations. Given the growing interest in these interventions, it is important to also address the role played by personality in determining positive attitudes toward them, since more than 47% of those who choose to undergo cosmetic surgery are likely to meet the criteria for a diagnosis of a specific mental disorder (Sarwer et al., 2004; Harth and Hermes, 2007; Latham, 2010). One of the disorders frequently associated with an interest in cosmetic surgery is histrionic personality disorder, this predisposition being explained by the unrealistic expectations, excessive emotionality and need for attention that characterize the histrionic (Gazize and Gharadaghi, 2013; Rashidi et al., 2022). The histrionic resorts to such interventions (which are sometimes unnecessary) every time he perceives a small defect in the body (AlaviHejazi et al., 2017; Nakamura and Koo, 2021). Another disorder that is related to the acceptance of cosmetic surgery is obsessive-compulsive personality disorder (Barahmand et al., 2010; Ramos et al., 2019), characterized by the paying of excessive attention to details, rules and organization, and when OCD people follow such a procedure we see unrealistic and extremely optimistic expectations about the results, a fixation on minor flaws and the desire to discover as much information as possible about the intervention in an attempt to control the whole experience (Camsari and Jowsey-Gregoire, 2019; Özkur et al., 2020). In addition to the two previously mentioned, borderline personality disorder too – characterized by impulsivity, emotional and relational instability, and fluctuations in self-image (Napoleon, 1993) – is associated with interest in cosmetic surgery (Morioka and Ohkubo, 2014; Vizgaitis and Lenzenweger, 2019); in a study by Morioka et al. (2014), a patient injured her upper eyelids following the operation, in a moment of impulsivity and due to abandonment anxiety. The most common personality disorder associated with the desire to undergo cosmetic surgery is, however, narcissistic personality disorder (Zojaji et al., 2014; Golshani et al., 2016). As a rule, people diagnosed with this disorder have a favorable attitude toward cosmetic surgery due to their belief that it will make them more attractive and consequently give them a better social, relational and romantic status (Ricci et al., 2010; Mohammadzadeh, 2014; Nakamura and Koo, 2017).

Regarding the Big Five model of personality, studies have shown that neuroticism is associated with low body esteem (Swami et al., 2013), emotional stability is negatively correlated with an unfavorable physical appearance evaluation (Kvalem et al., 2006), and also with self-objectification (Miner-Rubino et al., 2002). Self-objectification was associated with both agreeableness (Nweke et al., 2021), openness to experience (Rollero and De Piccoli, 2017), and neuroticism (Carrotte and Anderson, 2018). In Benford and Swami’s (2014) study, higher levels of neuroticism predicted male muscularity and higher levels of extraversion as well as lower levels of neuroticism predicted body esteem. Also, the longitudinal study conducted by Hartmann and Siegrist (2015) showed that personality traits are associated over time with the perception of body size as follows: women with a high score on the neuroticism dimension perceived themselves as fatter, while women and men scoring high on the conscientiousness dimension considered their body to be thinner over time. The Big Five traits were also associated with affective experiences (Wilt and Revelle, 2019; Barford et al., 2020), aspects that we can assume are related to body image dimensions. Given that the traits in the Big Five model were associated with factors that underlie the desire to undergo cosmetic surgery, we can assume that they may, in turn, represent key elements in making this decision. The Big Five model has demonstrated strong predictive validity about attitudes toward body size (Swami et al., 2008, 2010). However, the model was not related to the acceptance of cosmetic surgery in a specific way which is why there is no concrete conclusion in the specialized literature regarding the association between the two constructs. The present meta-analysis wants to address this limit.

Also, the personality traits proposed by the Dark Triad (narcissism, Machiavellianism, and psychoticism) were associated with aspects related to body image. Machiavellianism represents a risk factor and narcissism represents a protective factor regarding body image problems developed through self-objectification (Dryden and Anderson, 2019). Narcissism is also correlated with body checking (Waller et al., 2008), eating disorders (Brunton et al., 2005; Gordon and Dombeck, 2010), excessive exercise (Campbell and Waller, 2010; Miller and Mesagno, 2014; Cook et al., 2020) and muscle dysmorphia (Rodrigue et al., 2018; Boulter and Sandgren, 2022). Considering body modification, the study by Stanescu and Romascanu (2020) mentions increased levels of the three personality traits among people who have tattoos compared to those who do not, with psychoticism being the trait most likely to be associated with different types of bodily changes (Nathanson et al., 2006). The study by Kozlowska et al. (2023) supports the increased levels of narcissism, Machiavellianism, and psychopathy in people who are short and suffer from the Napoleon complex, as well as a desire to change certain body parts to be taller. Following the above, we can assume that personality traits may be factors of choice when people want to follow cosmetic surgery. However, few studies have investigated this association among the non-clinical population, so there is no consistent conclusion yet.

Along with the personality types described by the Dark Triad and Big Five theories and frequently associated in the specialist literature with the desire to undergo cosmetic surgery, other traits that do not necessarily belong to a specific and well-known theoretical model have been studied recently in connection with the probability that a person will opt for esthetic surgery, along with other variables that are crucial in clarifying the reasons why people resort to such procedures.

One of these traits is appearance-based rejection sensitivity, defined as a dispositional tendency that involves exaggerated reactions by people when they believe they will be rejected on the basis of their physical appearance (Park, 2007). Researchers have tried to show the importance of this trait in the development of attitudes toward cosmetic surgery, as well as in the appearance of some symptoms that have over time been correlated with the desire to undergo an esthetic operation: body dysmorphic disorder (Kelly et al., 2014), negative body image (Calogero et al., 2010), and unhealthy investment in physical appearance (Cash et al., 2004). Additionally, Henderson-King and Henderson-King (2005) highlight people’s fear of having an unattractive physique as a motivating factor in the desire to undergo an esthetic operation, while Sarwer et al. (2005) show the importance of messages received from others regarding body appearance (negative and mean comments by family or peers) in people’s decision to undergo cosmetic surgery. Given all this information, it is likely that a person who scores high on this personality trait will be more eager to change certain body parts they find unattractive as a result of messages received from others. Precisely for this reason, it is essential to broaden our knowledge of the relationship between appearance-based rejection sensitivity and the desire to have an esthetic operation, as there is currently no clear conclusion regarding the association between the two variables.

Another relevant trait is perfectionism. This personality trait was conceptualized by Hewitt and Flett (1991) as having three dimensions: self-oriented perfectionism, other-oriented perfectionism, and socially prescribed perfectionism. The same authors later focused on a new feature related to perfectionism, namely perfectionistic self-presentation, which in its turn is formed of three stable dimensions: perfectionistic self-promotion, non-disclosure of imperfection, and non-display of imperfection (Hewitt et al., 2003a,b). Research shows that perfectionists tend to have a negative body image due to the discrepancy between the actual and the desired image (Grammas and Schwartz, 2009; Barnett and Sharp, 2016; Teixeira et al., 2016), which may lead to a desire to undergo esthetic surgery to modify certain parts of the body that they consider imperfect (Hewitt et al., 2003a,b). Moreover, physical attractiveness tends to be associated with social relationships (Krebs and Adinolfi, 1975), so perfectionists may follow an esthetic procedure in order to gain social acceptance. In addition to these, other variables that have been correlated with interest in cosmetic surgery, such as eating disorders, excessive gym use, social physique anxiety, and body dysmorphic disorder have also been associated with perfectionism (Forbush et al., 2007; Arji et al., 2016; Mavrandrea and Gonidakis, 2022; Abdollahi et al., 2023). Thus, in light of this information, we can assume that there is a link between perfectionism and the desire to undergo an esthetic operation, but this link has not been sufficiently studied or reported in the specialist literature for a definite conclusion to be formulated. It is imperative, therefore, that in addition to the well-known theories of personality, other individual traits should be taken into consideration in any discussion of people’s interest in cosmetic surgery. Conceptualizing a model that contains the personality traits most often associated with cosmetic surgery would allow the development of guidelines to help evaluate patients so that results can be beneficial as possible for them.

We can thus observe that people who want to undergo an esthetic intervention may have various psychological characteristics that can affect their taking of such a decision. It is therefore recommended that they be evaluated by cosmetic surgeons armed with relevant information, with a view to the identification of any personality disorder. The surgeon will then know exactly how to relate to them (Sohrabi, 2011; Veer et al., 2014). Although the prevalence of mental disorders among people who wish to undergo cosmetic surgery is a high one, it is important to find out what personality factors underlie such a decision in the case of the non-clinical population as well. Cosmetic surgeons need to be able to identify people with particular psychological conditions, behavioral patterns or life situations (Adamson and Chen, 2008) that could negatively affect results obtained from the cosmetic surgery. When they have all this information, they will be much better prepared to provide personalized medical services that match the particularities of each candidate.

Although there are studies that investigate the link between personality traits and interest in having an esthetic operation, none of those so far seen by the authors gives a quantitative meta-analysis that looks specifically at the relationship between these variables. Thus, by carrying out this systematic review and the present meta-analysis, we aim to (1) synthesize the results obtained up to now by researchers regarding the abovementioned relationships and (2) analyze their scale. Identifying these relationships will allow esthetic surgeons to understand both the mechanisms underlying this decision and the need for psychological consultation before a person undergoes such a procedure. It will also allow psychotherapists to carry out intervention programs to help the people in question make the most beneficial decision for their physical and mental well-being. Furthermore, knowing the existence of the association between different kinds of personality traits (which are not necessarily related to a personality disorder) and acceptance of cosmetic surgery can be the starting point for defining future studies that are based on predictions or analyze causal relationships. In this way, the practitioners will know how to use a personalized approach with every patient based on his personality. Also, significant associations between personality traits and interest to undergo cosmetic surgery can encourage the implementation of treatment protocols based on patients’ personality. We used PRISMA guidelines in order to answer these research questions (Page et al., 2020).

## Methodology

### Eligibility criteria

A series of criteria were applied in order for studies to be considered eligible for inclusion in the meta-analysis. Thus, in terms of **research design**, only those that reported Pearson correlations between interest in undergoing an esthetic operation and a particular personality trait were included. At the same time, only studies that had a cross-sectional or longitudinal design were chosen. In the case of the longitudinal ones, when the effect sizes were reported, only those presented after the first evaluation of the variables of interest were included in the analysis.

The eligibility criterion used in the case of the **personality variable** proposed the inclusion of studies that operationalized this construct by referring to personality traits from different theories (e.g., Big Five, Dark Triad, Eysenck’s theory), traits recently investigated in the literature in the field (e.g., appearance-based rejection sensitivity) and personality traits not associated with a particular theory (e.g., perfectionism). Thus, studies that used instruments that measured personality or previously mentioned close concepts were considered eligible.

With regard to the variable **interest in undergoing an** esthetic **operation**, studies that used instruments that measured the following constructs were considered eligible: interest in/likelihood of undergoing an esthetic operation, acceptance of esthetic operations, consideration of cosmetic surgery.

As for the eligibility criteria associated with **participants**, they needed not to have had any previous cosmetic surgery and not to have bene diagnosed with a personality disorder. An age criterion was not included, as this phenomenon can also occur among adolescents, so the studies selected reported on participants of all age groups.

### Search strategies

The identification of research that met the eligibility criteria was carried out with the help of electronic searches in the following databases: ScienceDirect, PsycInfo, Web of Science, Scopus, Springer and PubMed. The following search algorithm was applied to the abstracts, titles and keywords: (“cosmetic surgery” OR “consideration of cosmetic surgery” OR “acceptance of cosmetic surgery” OR “interest in cosmetic surgery”) AND (personality OR “personality traits” OR “traits” OR “personality theory”). The studies chosen had all been published in English and subjected to a peer-review process. There was no specific time frame for the inclusion of studies; consideration was given to all studies published up until April 2023. The electronic search was complemented by a manual search, taking into account the references in the systematic reviews elaborated on topics of interest similar to that of the present meta-analysis. All articles identified were put into a list, from which the selection process was carried out.

### The selection process

**Figure 1:**
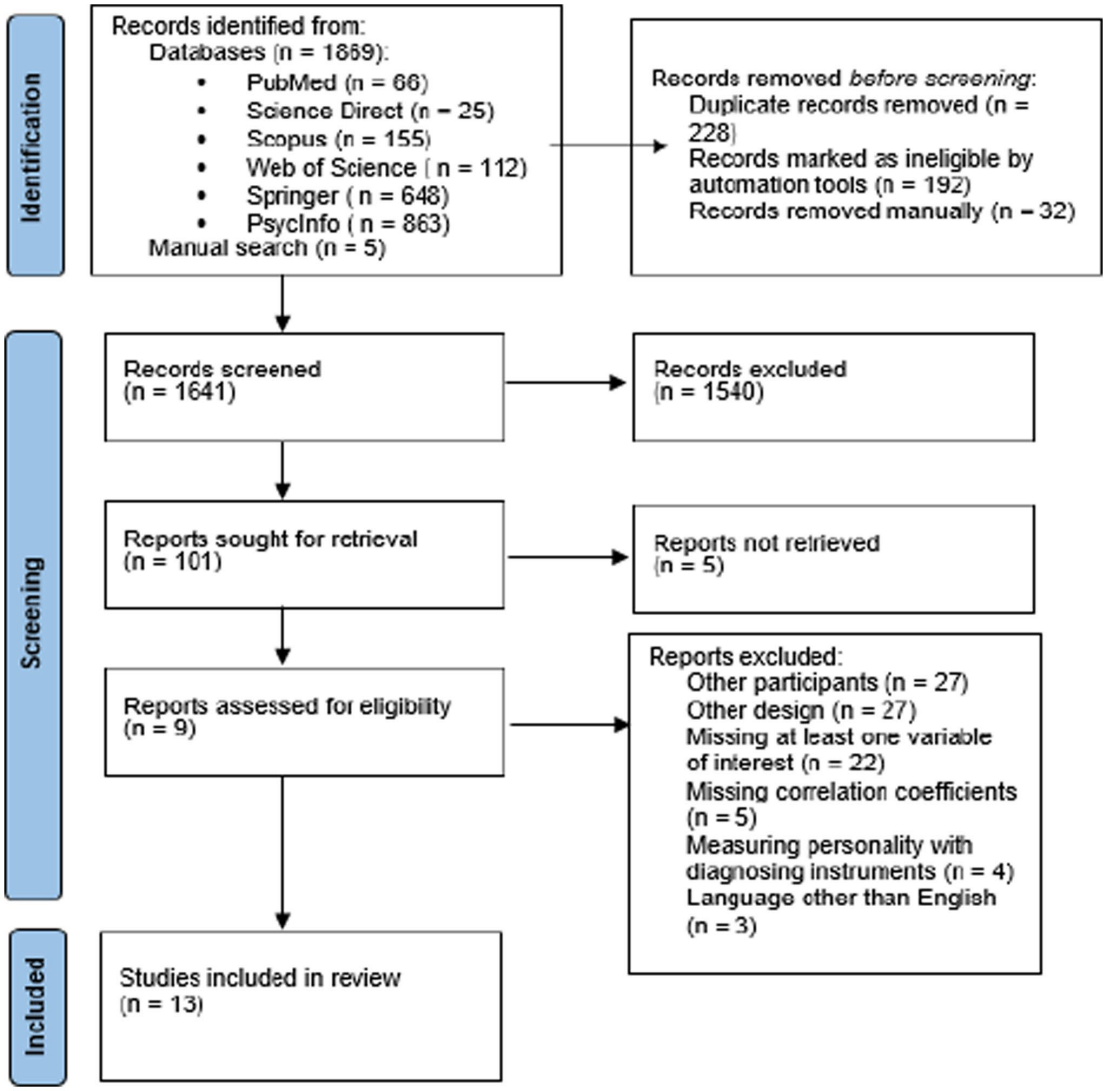
PRISMA flowchart illustrating article selection and inclusion.

One thousand eight hundred sixty-nine studies were identified from the electronic database search, and 5 five additional studies were picked out using the manual search based on information provided by the systematic reviews of Bascarane et al. (2021), Herruer et al. (2015), and Milothridis et al. (2016). Of the 1869 studies, 228 were eliminated as duplicates. The remaining 1,641 studies were subjected to a review of titles and abstracts, leading to 1,540 of them being eliminated. This elimination of studies based on the reading of titles and abstracts left us with 101 studies to be read in full. Of these, 5 did not allow full-text access. Of the full-text articles, 88 studies were eliminated, for the following reasons: 4 measured personality using instruments that sought to diagnose a specific pathology, 3 were in a language other than English (2 in Portuguese and 1 in Spanish), 5 performed different types of regression and did not report correlation analyses, 22 did not include both variables of interest but only one of them (4 did not investigate interest in cosmetic surgery), 18 did not investigate personality traits, 27 involved relevant categories of participants (20 had people diagnosed with personality disorders, 5 had people who had already undergone surgery, 1 investigated drag queens, and 1 looked at doctors), and 27 did not have the necessary design (3 systematic reviews, 7 comparative studies, 1 case study, 1 descriptive study, 1 retrospective study, 1 qualitative study, 12 theoretical studies, 1 letter & viewpoint). This procedure left 13 studies for inclusion in the meta-analysis stage of the research. The selection of articles was carried out separately by the two authors and any differences of opinion were debated until agreement was reached. Authors of studies for which access to data could not be obtained were contacted by email, but no response has been received to date. The PRISMA flowchart (Figure 1) shows the full process of article selection (Page et al., 2020).

### Data extraction

To perform the meta-analytic statistical analysis, the necessary data were extracted from each study included in the final meta-analysis (correlation coefficient and number of participants). Information was also extracted regarding the objectives of the studies, the main tools used, the characteristics of the participants and the results obtained.

### The meta-analytic strategy

The meta-analysis was performed using the Comprehensive Meta-Analysis program, version 4 (Borenstein et al., 2022). With regard to effect size, in this paper the Bravais-Pearson correlation coefficient (*r*) was reported. The Pearson correlation coefficient was measured within the relationships between personality, operationalized in all its previously mentioned forms, and interest in undergoing an esthetic operation. As a statistical approach, a **random effects meta-analytic model** was proposed, because there is a fairly wide variation among studies both in terms of their methodology for evaluating variables (different evaluation tools were used, and in different ways; for example, for measuring interest in having an esthetic operation, some authors used the ACSS scale and gave a total score for it, while others gave a score for each subscale, or used only one subscale within the instrument) and also with regard to the characteristics of the participants (most studies had female participants, some used both female and male participants, and others had adolescent or young adult participants). Despite this, Hedges and Vevea (1998) nevertheless recommend adopting this statistical approach, since it offers a greater degree of generalizability of results than the fixed effects model. The use of this analytical model, using as effect size the correlation coefficient *r*, resorts to the transformation of the coefficient into Fischer’s z in order to perform the meta-analytical calculation; after the calculating operation, the result obtained is returned to its initial form (that is, it is transformed back into *r*). This process occurs because when we use Fischer’s z the dispersion of each effect size index depends exclusively on sample size, whereas when we use *r* it is interdependent with both sample size and correlation size. At the same time, if we are talking about empirical studies, we can see that the sample size is represented by the number of participants (N). If we are talking about the meta-analytic approach, the sample size is given by the number of studies included (k), so that in this meta-analysis the assessment of inter-study heterogeneity was calculated using the *I*^2^ index. Attempts were made to apply meta-regressions to investigate the moderating nature of various variables, but the small number of studies did not allow this analysis.

### Sources of distortion

Egger’s intercept (Egger et al., 1997) was calculated to see if there was any publication bias. Regarding the bias assessment for the studies included in the final analysis we used the STROBE checklist for observational studies (von Elm et al., 2007).

## The results of the systematic review

Regarding the relationship between personality traits and readiness to undergo cosmetic surgery, Vally et al. (2020) showed that the need for uniqueness (associated with openness to experience) was associated with body appreciation and interest in cosmetic surgery, while investment in physical appearance was associated with body appreciation, need for uniqueness, and interest in cosmetic surgery. Jávo et al. (2012) note that only emotional stability is negatively correlated with interest in cosmetic surgery among people without an eating disorder, while extraversion, agreeableness, openness to experience, and conscientiousness are not associated with cosmetic surgery. This is supported by the study of Mowen et al. (2009), which demonstrates a significant link between the neuroticism dimension and attitudes toward cosmetic surgery. The study of Swami et al. (2009) shows that the gender and age of participants are associated with willingness to undergo cosmetic surgery through the mediating role of personality traits, conformity (associated with low openness to experience) and self-esteem. Leitermann et al. (2016) show that there are no significant associations between the extraversion-introversion dimension and acceptance of cosmetic surgery.

If we focus on the appearance-based rejection sensitivity personality trait [conceptualized by Park (2007) as a personality trait; personal rejection sensitivity, which is related to this variable, is just a simple trait, according to Downey and Feldman (1996)], Calogero et al. (2010), Park et al. (2009), and Park et al. (2010) have all shown that this is a good predictor of future acceptance of cosmetic surgery. At the same time, Naami and Mahmood Salehi (2016), Sherry et al. (2004), and Swami and Mammadova (2012) showed that perfectionism as an operationalized personality trait in all of its forms (Hewitt and Flett, 1991) predicts interest in undergoing an esthetic procedure. When we turn to the personality traits of the Dark Triad model, we can see that narcissism is not correlated with the desire to undergo cosmetic surgery (Kalantar-Hormozi et al., 2016; Chegeni and Atari, 2017); the traits in this model that are associated with such interventions are psychopathy and Machiavellianism (Chegeni and Atari, 2017).

A summary of the characteristics of the studies included in the review is given in Table 1.

**Table 1:** Synthesis of the characteristics of the studies included in the systematic review (*k* = 13).

Study	Objective	Design	Participant characteristics (number, mean age, male number, female number, participant type)	Instruments		Results
				VI	VD	
Vally et al. (2020) (United Arab Emirates)	Investigation of association between body appreciation and self-expression, such as uniqueness, interest in cosmetic surgery and investment in appearance	Cross-sectional	Participant number: 256 Mean age: 20.35 Men number: 0 Women number: 256 Participant type: University students	The self-attributed need for uniqueness scale (SANU) Uniqueness trait was related to Openness dimension (from Big Five model) – the unconventional trait; resistance to culturally imposed ideals of beauty	Six items from the interest in cosmetic surgery scale (ICSS)	Need for uniqueness was associated with body appreciation and interest in cosmetic surgery. Investment in a distinctive appearance was also associated with body appreciation, need for uniqueness and interest in cosmetic surgery.
Park et al. (2009) (USA)	Investigation of association between appearance-based rejection sensitivity and acceptance of cosmetic surgery	Cross-sectional	Participant number: 133 Mean age: 19.15 Men number: 61 Women number: 72 Participant type: College students	Appearance-RS Scale Appearance-based rejection sensitivity was conceptualized as a stable personality trait	Three items adapted from Acceptance of Cosmetic Surgery Scale (ACSS)	Association between personality traits and situation is important when predicting cosmetic surgery interest. Appearance-based rejection sensitivity was correlated with the desire for cosmetic surgery.
Jávo et al. (2012) (Norway)	The examination of predictors of an interest in cosmetic surgery among women with and without eating disorders	Cross-sectional	Participant number: 1861 Mean age: 28 Men number: 0 Women number: 1861 Participant type: Women with and without eating disorders	10-item version of the Big Five Personality Inventory	Questionnaire in which the applicants reported an interest in cosmetic surgery	Interest in liposuction was predicted by various factors including personality traits
Sherry et al. (2004) (Canada)	Investigation of perfectionism trait related to acceptance of cosmetic surgery	Cross-sectional	Participant number: 292/736/95 Mean age: 19.52/19.58/27.49 Men number: 0/209/52 Women number: 292/527/43 Participant type: University students/Young adults	First study: Subscales of Perfectionistic Self-Presentation Scale (PSPS) Second and third studies: Subscales of Multidimensional Perfectionism Scale (MPS)	Single-item measure in both studies: “Have you ever thought about having cosmetic surgery done?”	Perfectionists tend to pursue appearance ideals and are more likely to undergo cosmetic surgeries.
Swami et al. (2009) (UK)	Investigation of association between personality traits, demographics, individual differences and acceptance of cosmetic surgery	Cross-sectional	Participant number: 332 Mean age: 24.72 Men number: 131 Women number: 201 Participant type: University students	TIPI (Ten Item Personality Inventory)	ACSS (Acceptance of cosmetic surgery scale)	Sex and age were associated with acceptance of cosmetic surgery through the mediating influences of personality traits, self-esteem and conformity.
Leitermann et al. (2016) (Germany)	Investigation of individual attitudes of young adults regarding the acceptance of cosmetic surgeries	Cross-sectional	Participant number: 104 Mean age: 30 Men number: 23 Women number: 81 Participant type: Young adults	Items of FPI-R (Freiburger Personality Inventory)	Questions about body dissatisfaction and the desire to change certain parts by receiving cosmetic treatment	There were no significant correlations between extraversion-introversion personality dimensions and acceptance of cosmetic surgery
Mowen et al. (2009) (USA)	Investigation of association between personality traits, tanning propensity, and acceptance of cosmetic surgery	Cross-sectional	Participant number: 231 Mean age: 49.2 Men number: 109 Women number: 122 Participant type: Adults	Measure created by authors for this study	Measure created by authors for this study	Regarding personality traits, neuroticism was positively associated with a desire to undergo cosmetic surgery
Chegeni and Atari (2017) (Iran)	Investigation of the association between Dark Triad Personality and acceptance of cosmetic surgery	Cross-sectional	Participant number: 222 Mean age: 24.59 Men number: 102 Women number: 120 Participant type: University students	The Short Dark Triad (SD3)	ACSS (Acceptance of cosmetic surgery scale) – Consider subscale	Psychopathy was associated with acceptance of cosmetic surgery both in women and men. Machiavellianism was associated with interest in cosmetic surgery only in women. There was no significant association between narcissism and considering cosmetic surgery.
Swami and Mammadova (2012) (UK)	Examination of the relationship between cosmetic surgery, perfectionism trait dimmensions, appearance schemas, relationship satisfaction, love styles and reassurance seeking	Cross-sectional	Participant number: 300 Mean age: 23.17 Men number: 0 Women number: 300 Participant type: Adults	Subscales of Perfectionist Self-Presentation Scale (PSPS)	ACSS (Acceptance of cosmetic surgery scale) – Consider subscale	Perfectionism dimensions predict consideration of cosmetic surgery.
Naami and Mahmood Salehi (2016) (Iran)	Investigation of the relationship between cosmetic surgery interest, personality traits, perfectionism, mindfulness and mental health.	Cross-sectional	Participant number: 200 Mean age: 24.59 Men number: 0 Women number: 200 Participant type: High school females	Multidimensional Perfectionism Scale (MPS) NEO-PI-R	Acceptance of Cosmetic Surgery Scale (ACSS)	Results showed that all the predictors investigated were related with interest in cosmetic surgery among women.
Kalantar-Hormozi et al. (2016) (Iran)	Investigation of the relationship between self-esteem, narcissism, self-perceived attractiveness and interest in cosmetic surgery	Cross-sectional	Participant number: 300 Mean age: 25.97 Men number: 0 Women number: 300 Participant type: University students	Single-Item Narcissism Scale (SINS)	ACSS (Acceptance of cosmetic surgery scale) – Consider subscale	Self-esteem and self-perceived attractiveness predict attitudes toward cosmetic surgery. There was no significant association between narcissism and considering cosmetic surgery.
Park et al. (2010) (USA)	Investigation of the association between appearance-based rejection sensitivity, body dysmorphic disorder and interest in cosmetic surgery	Cross-sectional	Participant number: 349 Mean age: 19.30 Men number: 128 Women number: 229 Participant type: College students	The original Appearance-based rejection sensitivity scale	Acceptance of Cosmetic Surgery Scale (ACSS)	Results showed that appearance-based rejection sensitivity predicts body dysmorphic disorder and acceptance of cosmetic surgery for both intrapersonal and social reasons.
Calogero et al. (2010) (UK)	Investigation of the association between appearance-based rejection sensitivity, body dysmorphic disorder and interest in cosmetic surgery	Cross-sectional	Participant number: 106 Mean age: 21.54 Men number: 24 Women number: 82 Participant type: University students	Appearance-based rejection sensitivity scale	Acceptance of Cosmetic Surgery Scale (ACSS)	Results showed that appearance-based rejection sensitivity trait predicts the acceptance of cosmetic surgery and greater consideration of having this type of intervention in the future.

## Meta-analysis results

### The relationship between personality traits and interest in undergoing cosmetic surgery (Big Five model)

Regarding the relationship between personality traits operationalized according to the Big Five model and interest in having cosmetic surgery, the results only partially confirmed the researchers’ expectations (it had been assumed that there was a relationship between each of the personality traits and interest in undergoing cosmetic surgery).

**Figure 2:**
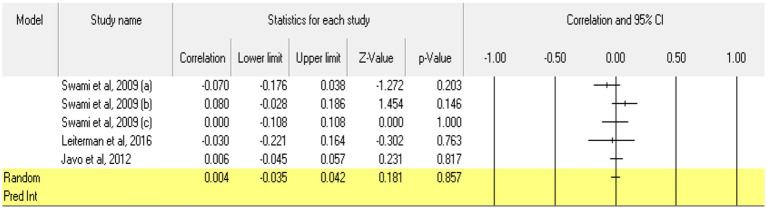
Associations reported between extraversion and acceptance of cosmetic surgery.

**Figure 3:**
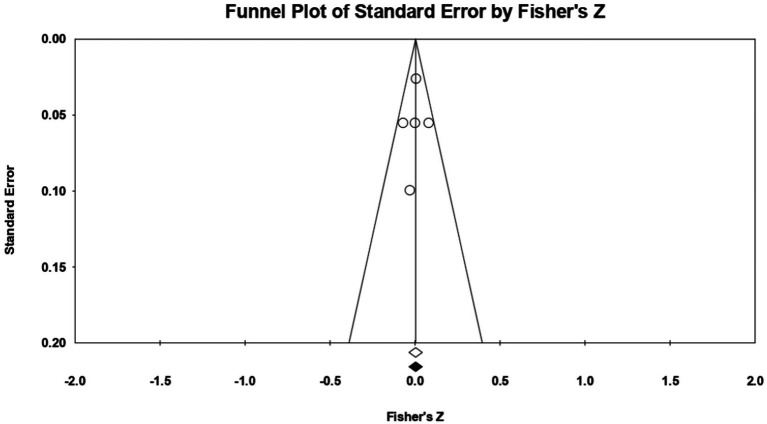
Funnel plot regarding bias publication for studies reporting an association between extraversion and acceptance of cosmetic surgery.

Taking **extraversion** as the first trait, 5 relationships were extracted from 4 eligible studies (those which measured extraversion as a personality trait; from the study by Mowen et al., 2009, we could not extract relationships existing between extraversion and acceptance of cosmetic surgery, because Mowen only reported the relationship between introversion and the acceptance of esthetic operations; since there are no other studies of the introversion dimension, a meta-analytic approach could not be taken here). The analysis revealed a statistically insignificant *r* correlation coefficient, with a value of 0.004 [−0.035; 0.0342], *p* > 0.05, *p* = 0.857 (Figure 2). In this case, there is no marked heterogeneity in the observed effect sizes (*I*^2^ = 0.000), *p* > 0.05, *p* = 0.42, so there are no wide variations from one study to another. Regarding publication bias (Figure 3), Egger’s t test was not statistically significant (intercept = −0.292, *p* = 0.82). Thus, according to the results obtained, the hypothesis is not statistically supported.

**Figure 4:**
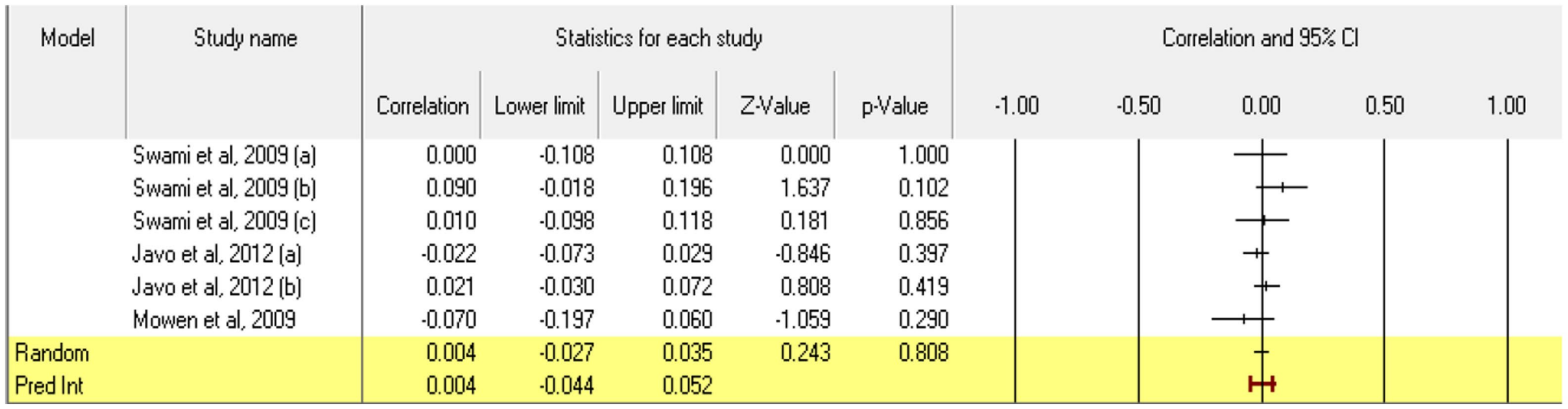
Associations reported between agreeableness and acceptance of cosmetic surgery.

**Figure 5:**
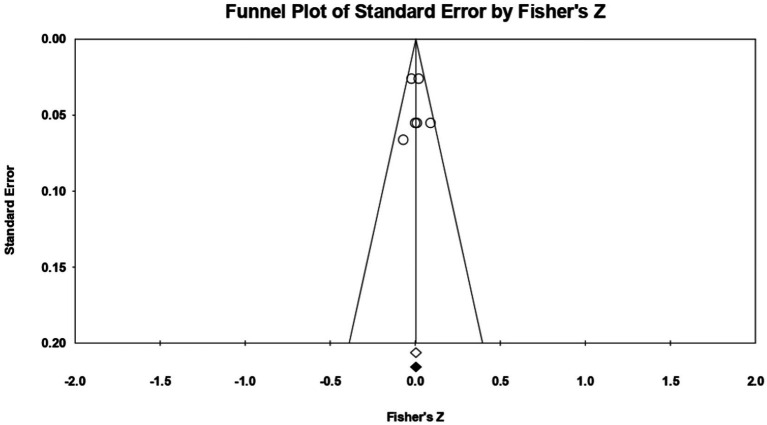
Funnel plot regarding bias publication for studies reporting an association between agreeableness and acceptance of cosmetic surgery.

Regarding **agreeableness**, 6 relationships were extracted from 3 eligible studies (those which measured agreeableness as a personality trait in accordance with the Big Five model); in addition to a high degree of agreeableness, the study by Mowen et al. (2009) also measured a low level of agreeableness (they evaluated competitiveness trait and since there are no other studies of this latter dimension, a meta-analytical approach to it was not possible). The analysis revealed a statistically insignificant *r* correlation coefficient, with a value of 0.004 [−0.027; 0.035], *p* > 0.05, *p* = 0.808 (Figure 4). In this case, there is no marked heterogeneity in the observed effect sizes (*I*^2^ = 2.817), *p* > 0.05, *p* = 0.39, so there are no wide variations from one study to another. Regarding publication bias (Figure 5), Egger’s *t* test was not statistically significant (intercept = 0.205, *p* = 0.87). Thus, according to the results obtained, the hypothesis is not statistically supported.

**Figure 6:**
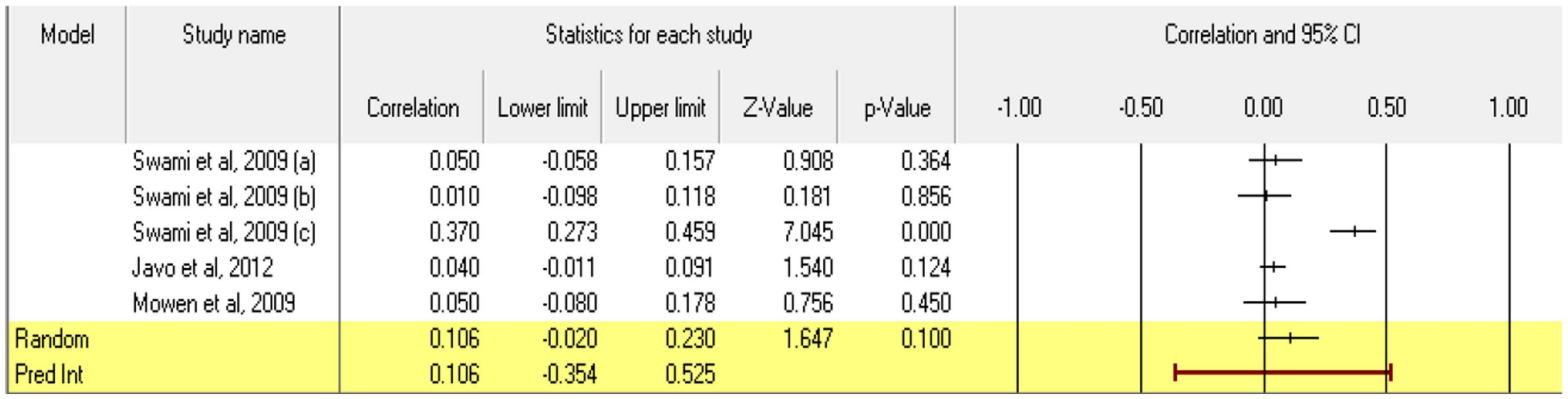
Associations reported between conscientiousness and acceptance of cosmetic surgery.

**Figure 7:**
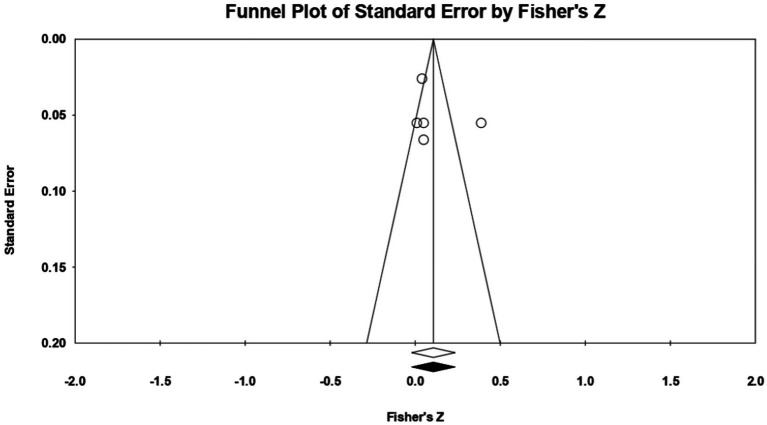
Funnel plot regarding bias publication for studies reporting an association between conscientiousness and acceptance of cosmetic surgery.

For **conscientiousness**, 5 relationships were extracted from 3 eligible studies (ones that measured both constructs in accordance with the eligibility criteria). The analysis revealed a statistically insignificant *r* correlation coefficient, with a value of 0.106 [−0.020; 0.230], *p* > 0.05, *p* = 0.10 (Figure 6). Calculation of the coefficient *I^2^* shows a marked heterogeneity in effect sizes (*I*^2^ = 88.818), *p* < 0.01. This wide variation is most likely due to the small number of studies, but also to their characteristics (the ways the variables are operationalized, the characteristics of the participants, the measuring instruments used). Regarding publication bias (Figure 7), Egger’s t test was not statistically significant (intercept = 2.506, *p* = 0.57). Thus, according to the results obtained, the hypothesis is not statistically supported.

**Figure 8:**
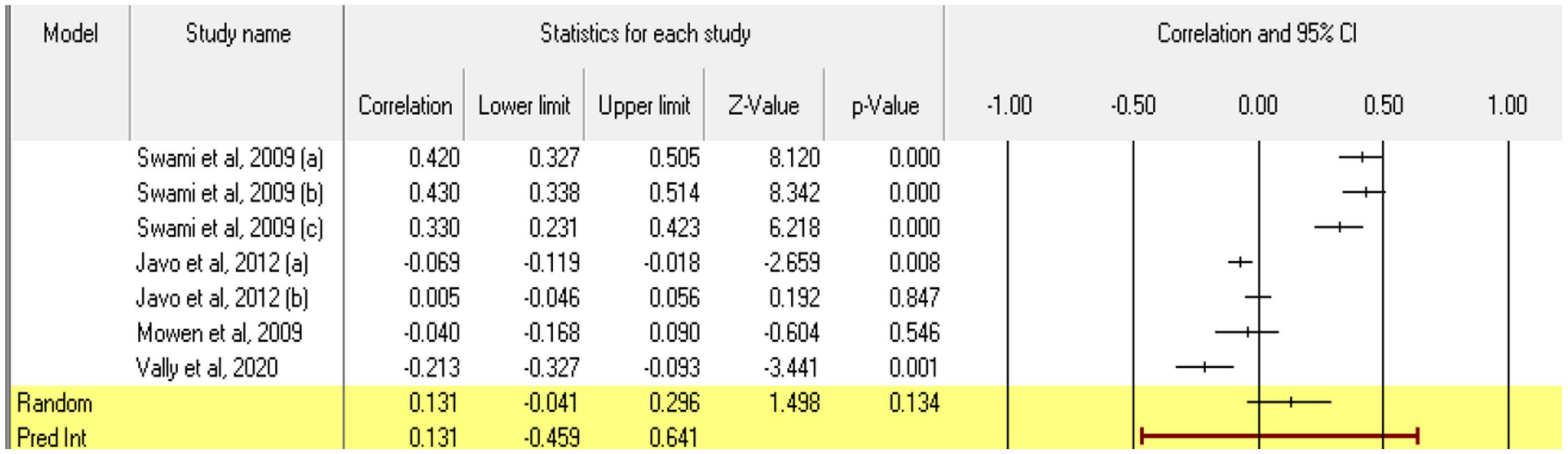
Associations reported between openness and acceptance of cosmetic surgery.

**Figure 9:**
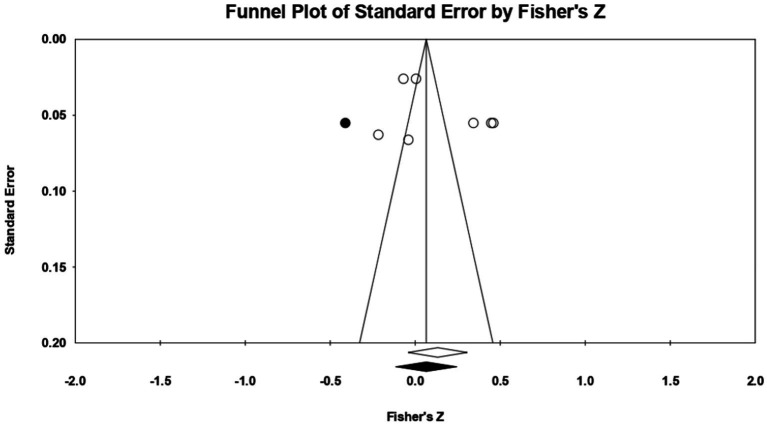
Funnel plot regarding bias publication for studies reporting an association between openness and acceptance of cosmetic surgery.

For **openness**, 7 relationships between the two constructs of interest were extracted from 4 eligible studies. The analysis revealed a statistically insignificant correlation coefficient *r*, with a value of 0.131 [−0.041; 0.296], *p* > 0.05, *p* = 0.13 (Figure 8). However, there is a marked heterogeneity between the observed effect sizes (*I^2^* = 96.950), *p* < 0.01, this wide variation being most likely due to the small number of studies, but also to their characteristics (the ways the variables are operationalized, the characteristics of the participants, the measuring instruments used). Regarding publication bias, Egger’s t test was not statistically significant (intercept = 2.50, *p* = 0.57) (Figure 9). In this case, the hypothesis was not supported.

**Figure 10:**
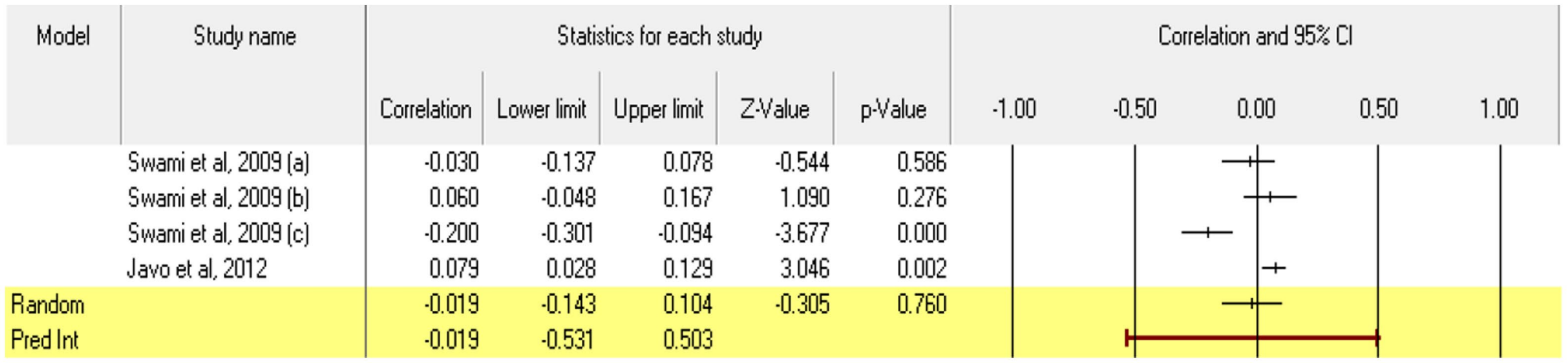
Associations reported between low neuroticism and acceptance of cosmetic surgery.

**Figure 11:**
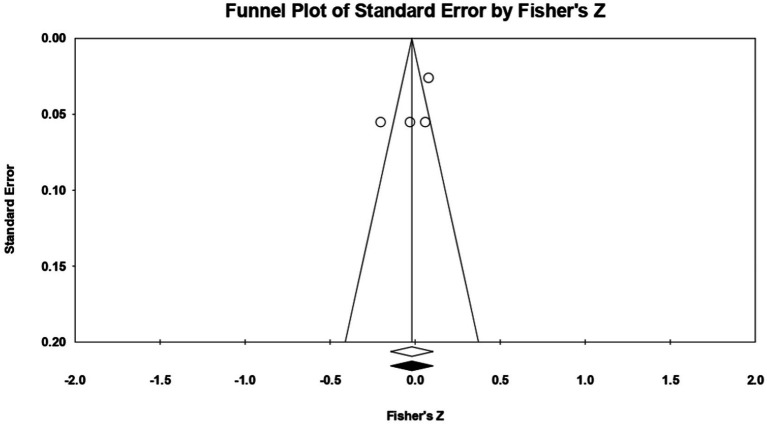
Funnel plot regarding bias publication for studies reporting an association between low neuroticism and acceptance of cosmetic surgery.

Turning to **low neuroticism** (or emotional stability) 4 relationships were extracted from two eligible studies [from the study of Mowen et al. (2009), we could not extract relationships between emotional stability and acceptance of cosmetic surgery, as it only reported the relationship between neuroticism and willingness to undergo an esthetic procedure; as there were no other studies on the dimension neuroticism, a meta-analytic approach could not be taken here]. The analysis revealed a statistically insignificant correlation coefficient *r*, with a value of −0.019 [−0.143; 0.104], *p* > 0.05, *p* = 0.76 (Figure 10). In this case, there is a marked heterogeneity in the observed effect sizes (*I*^2^ = 86.845), *p* < 0.01, this wide variation being most likely due to the small number of studies, but also to their characteristics (the ways the variables are operationalized, the characteristics of the participants, the measuring instruments used). Regarding publication bias, Egger’s t test was not statistically significant (intercept = 6.59, *p* = 0.24) (Figure 11). In this case, the hypothesis was not supported.

### The relationship between personality traits and interest in undergoing an esthetic procedure (Dark Triad model)

**Figure 12:**
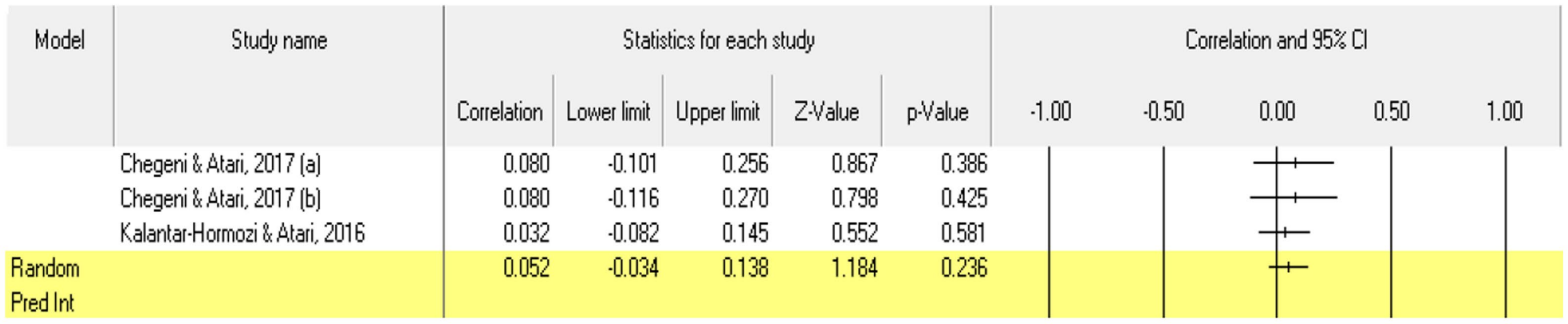
Associations reported between narcissism and acceptance of cosmetic surgery.

**Figure 13:**
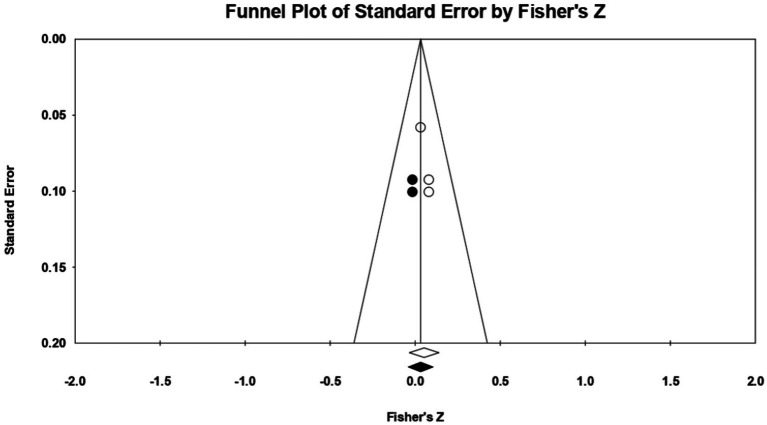
Funnel plot regarding bias publication for studies reporting an association between narcissism and acceptance of cosmetic surgery.

Looking at the traits highlighted by the Dark Triad model, a meta-analytical approach was carried out only for narcissism, as a personality trait, because in the cases of Machiavellianism and psychopathy not enough eligible studies/correlational relationships were recorded for us to be able to perform the statistical analysis (*k* = 2 for each of the two traits). Referring to **narcissism**, 3 relationships were extracted from the 2 eligible studies. The analysis revealed a statistically insignificant *r* correlation coefficient, with a value of 0.052 [−0.034; 0.138], *p* > 0.05, *p* = 0.23 (Figure 12). Following analysis, no marked heterogeneity in the observed effect sizes was noted (*I*^2^ = 0.000), *p* > 0.05, *p* = 0.86, so there are no wide variations from one study to another. Regarding publication bias, Egger’s *t*-test was not statistically significant (intercept = 1.23, *p* = 0.08) (Figure 13). In this case, the hypothesis was not supported.

### The relationship between appearance-based rejection sensitivity, as a stable personality trait, and interest in undergoing an esthetic operation

**Figure 14:**
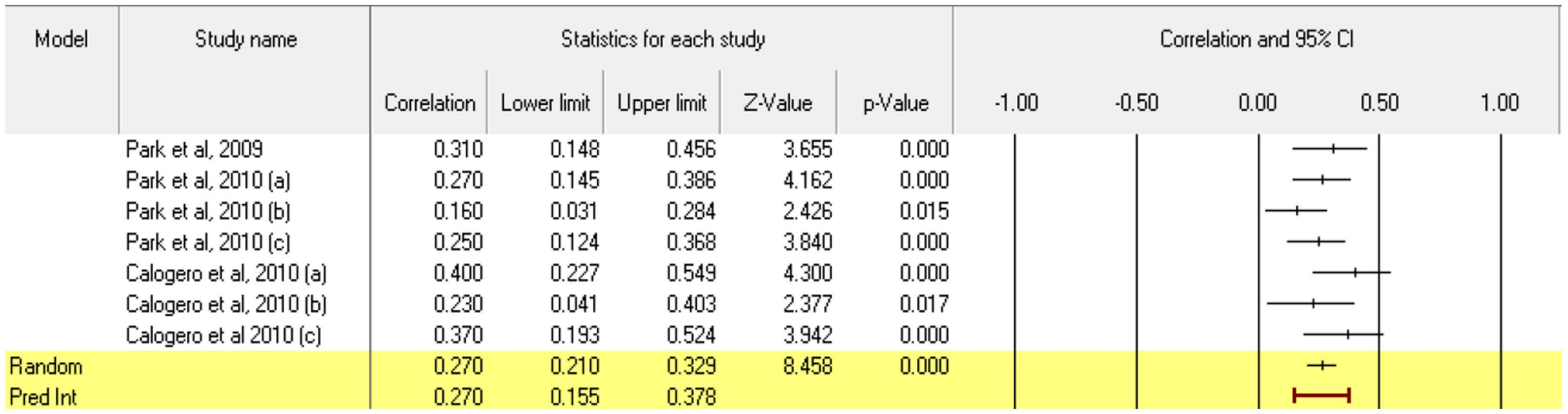
Associations reported between appearance-based rejection sensitivity and acceptance of cosmetic surgery.

**Figure 15:**
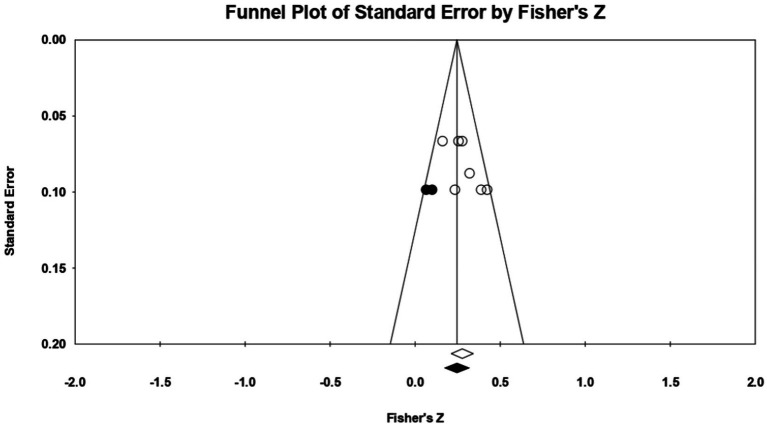
Funnel plot regarding bias publication for studies reporting an association between appearance-based rejection sensitivity and acceptance of cosmetic surgery.

In this case, 7 relationships were extracted from 3 eligible studies, so that a statistically significant correlation coefficient *r* was recorded, with a value of 0.270 [0.210; 0.329], *p* < 0.01 (Figure 14). Following analysis, no marked heterogeneity in the observed effect sizes was noted (*I*^2^ = 14.846), *p* > 0.05, *p* = 0.31, so there are no wide variations from one study to another. Regarding publication bias, Egger’s t-test was not statistically significant (intercept = 3.73, *p* = 0.09) (Figure 15). The hypothesis was supported in this case.

### The relationship between perfectionism, as a stable personality trait, and interest in undergoing an esthetic operation

In this meta-analysis, the perfectionism trait was conceptualized according the model by Hewitt and Flett (1991) as having three dimensions: **self-oriented perfectionism, other-oriented perfectionism**, and **socially prescribed perfectionism**. The same authors later focused on a new feature related to perfectionism, namely perfectionistic self-presentation, which in its turn is formed of three stable dimensions**: perfectionistic self-promotion, non-disclosure of imperfection**, and **non-display of imperfection** (Hewitt et al., 2003a,b).

**Figure 16:**
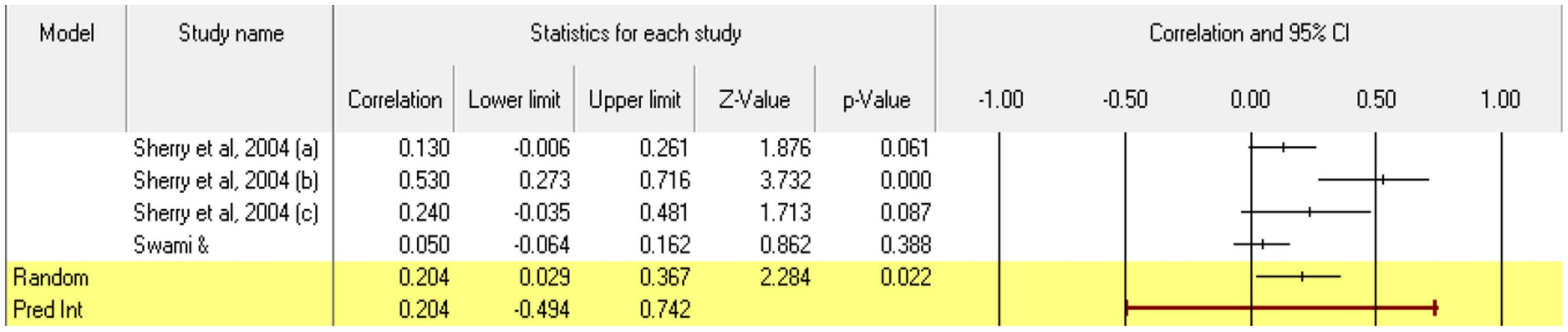
Associations reported between perfectionistic self-promotion and acceptance of cosmetic surgery.

**Figure 17:**
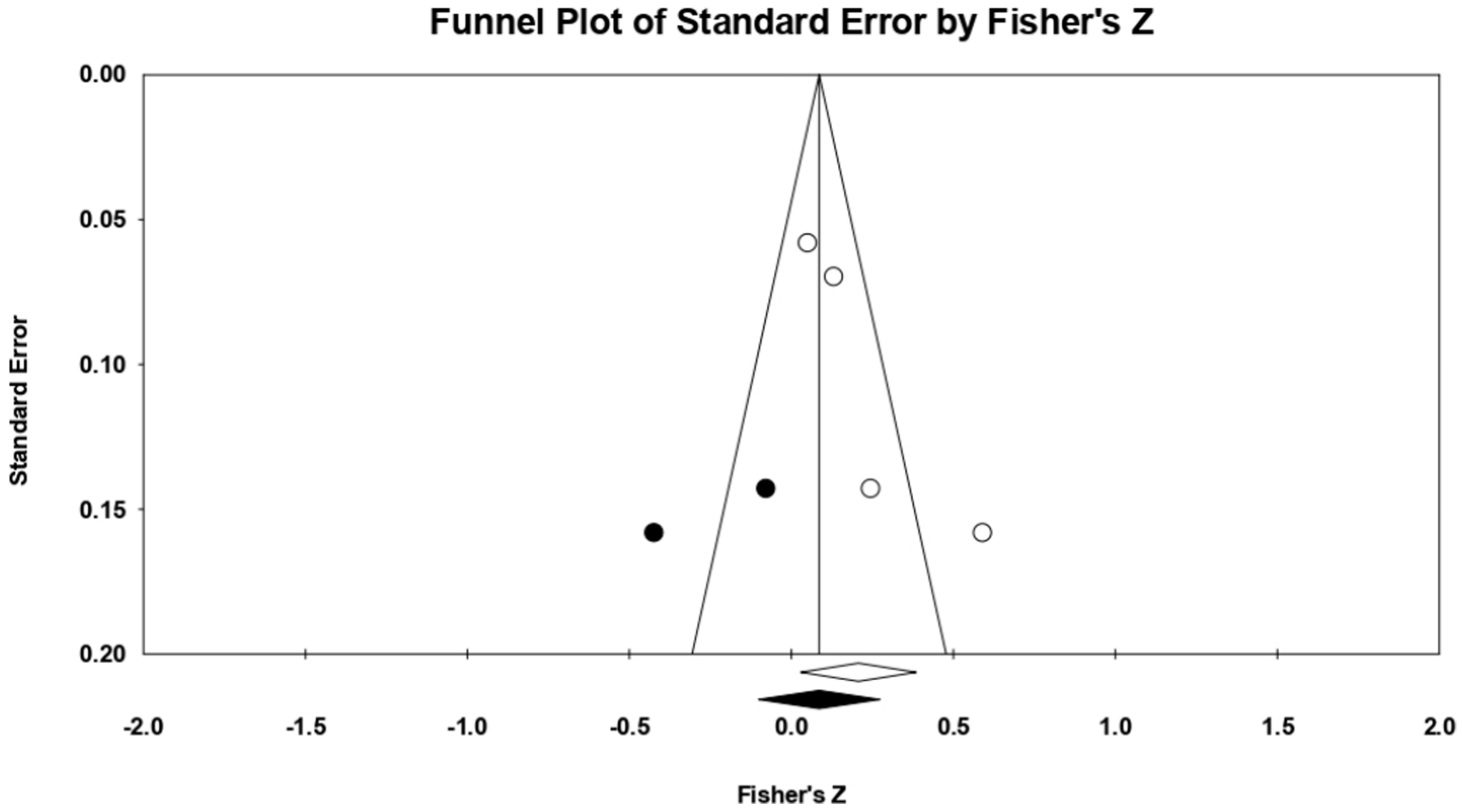
Funnel plot regarding bias publication for studies reporting an association between perfectionistic self-promotion and acceptance of cosmetic surgery.

Taking **perfectionistic self-promotion** as the first trait of the model, 4 relationships were extracted from 2 eligible studies. The analysis revealed a statistically significant *r* correlation coefficient, with a value of 0.204 [0.029; 0.367] *p* < 0.05, *p* = 0.02 (Figure 16). In this case, there is a marked heterogeneity in the observed effect sizes (*I*^2^ = 72.761), *p* < 0.05, *p* = 0.01, this wide variation being most likely due to the small number of studies, but also to their characteristics (the ways the variables are operationalized, the characteristics of the participants, the measuring instruments used). Regarding publication bias (Figure 17), Egger’s t test was not statistically significant (intercept = 3.901, *p* = 0.09). Thus, according to the results obtained, the hypothesis is statistically supported.

**Figure 18:**
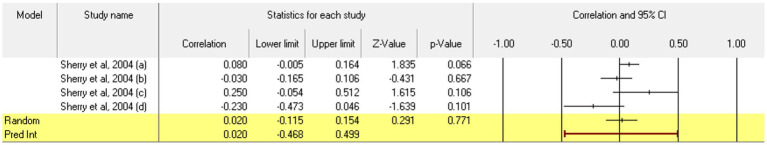
Associations reported between self-oriented perfectionism and acceptance of cosmetic surgery.

**Figure 19:**
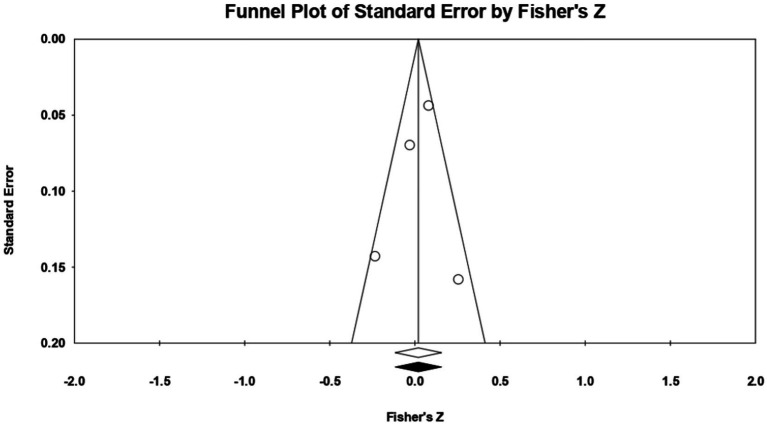
Funnel plot regarding bias publication for studies reporting an association between self-oriented perfectionism and acceptance of cosmetic surgery.

For **self-oriented perfectionism**, 4 relationships between the two constructs of interest were extracted from 1 eligible study. The analysis revealed a statistically insignificant correlation coefficient *r*, with a value of 0.020 [−0.115; 0.154], *p* > 0.05, *p* = 0.77 (Figure 18). Following analysis, no marked heterogeneity in the observed effect sizes was noted (*I*^2^ = 59.411), *p* > 0.05, *p* = 0.06, so there are no wide variations from one study to another. Regarding publication bias, Egger’s t-test was not statistically significant (intercept = −0.858, *p* = 0.70) (Figure 19). In this case, the hypothesis was not supported.

**Figure 20:**
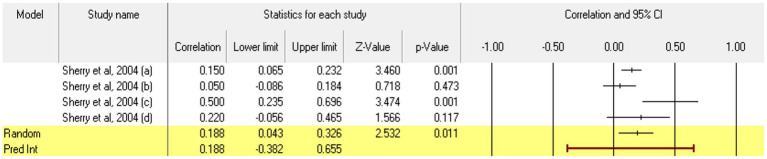
Associations reported between socially prescribed perfectionism and acceptance of cosmetic surgery.

**Figure 21:**
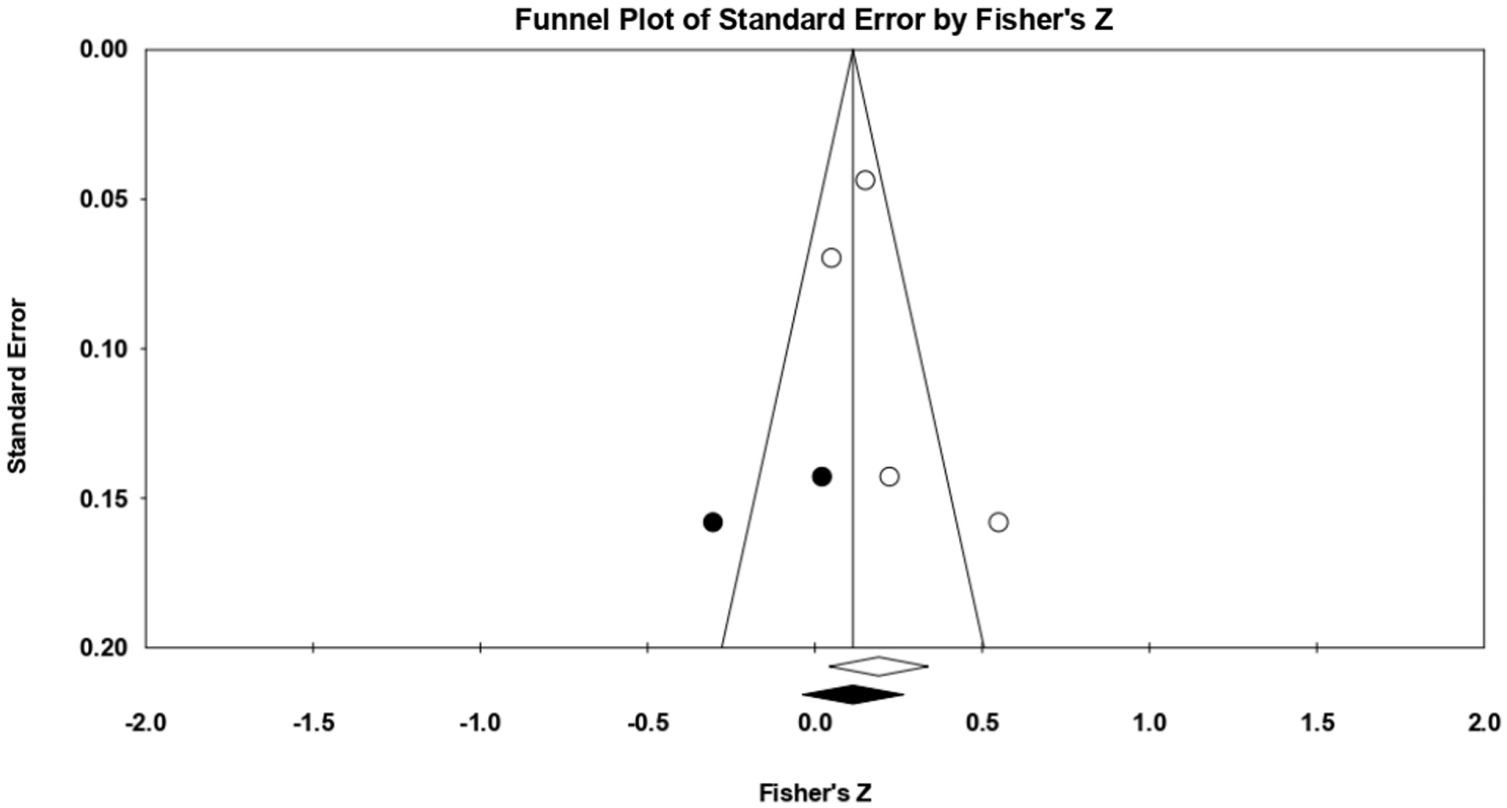
Funnel plot regarding bias publication for studies reporting an association between socially prescribed perfectionism and acceptance of cosmetic surgery.

For **socially prescribed perfectionism**, 4 relationships between the two constructs of interest were extracted from 1 eligible study. The analysis revealed a statistically significant correlation coefficient *r*, with a value of 0.188 [0.043; 0.326], *p* < 0.05, *p* = 0.01 (Figure 20). In this case, there is a marked heterogeneity in the observed effect sizes (*I*^2^ = 65.527), *p* < 0.05, *p* = 0.03, this wide variation being most likely due to the small number of studies, but also to their characteristics (the ways the variables are operationalized, the characteristics of the participants, the measuring instruments used). Regarding publication bias (Figure 21), Egger’s t test was not statistically significant (intercept = 1.888, *p* = 0.40). Thus, according to the results obtained, the hypothesis is statistically supported.

Regarding **non-disclosure of imperfection, non-display of imperfection, and other-oriented perfectionism** a meta-analytic approach could not be taken here, as there were no enough studies on these topics. Also, regarding **perfectionism** only one study conceptualized this variable as a single trait without any dimensions (Naami and Mahmood Salehi, 2016).

## The risk of bias

Regarding the bias assessment for the studies included in the final analysis we used the STROBE checklist for observational studies (von Elm et al., 2007). Each item included in the checklist was considered. We scored the items following the procedure used by Ştefǎnuţ et al. (2021) in their meta-analysis (when the study assessed the characteristics described by a specific item, it was noted with “0,” otherwise it was noted with “1”; for the items which required considering several characteristics, the value “1” was divided according to the number of those aspects. The risk of bias for each study was obtained by summing the results registered for every item). Twelve of the studies included in the final analysis reported a low risk of bias and one of them (Leitermann et al., 2016) reported a moderate risk of bias. The scores ranged between 1.5 (Chegeni and Atari, 2017) and 5.3 (Leitermann et al., 2016). The topics less considered included size sample calculation, approaching missing data and addressing possible sources of bias.

## Discussion

This meta-analysis aimed to study the relationship between interest in undergoing cosmetic surgery and personality traits, operationalized in the form of several theoretical models and dimensions. From what is known to date by the authors, this is the first meta-analysis to have focused on studying these variables of interest. To carry out the meta-analytic task, 13 scientific articles identified in the following databases were analyzed: ScienceDirect, PsycInfo, Web of Science, Scopus, Springer, and PubMed. Although the number of studies in the meta-analysis is relatively small, this research study has a number of strong points, most particularly the use of meta-analytical statistical techniques and the choice of rigorous criteria for the inclusion or exclusion of studies.

As previously noted, studies that had a cross-sectional design, the Big Five model and the Dark Triad as theoretical models were introduced. Studies that highlighted personality traits recently investigated in the specialist literature (perfectionism and appearance-based rejection sensitivity) were also introduced into the analysis. Both participant characteristics and research design characteristics were quite varied. The subjects of the studies ranged from teenagers to young adults to adults aged 35–55. In terms of design characteristics, a wide diversity of research instruments was observed (several studies used standardized measurement methods to measure all variables, others used a mix of instruments – some standardized, some non-standardized – and one used only non-standardized instruments). It was observed that the highest number of studies investigated were carried out in the USA, UK and Iran, followed by Norway, Germany, the UAE and Canada. Regarding the statistical analyses used in the studies that qualified for the meta-analysis, most of them used advanced techniques in order to better understand the relationship between the variables measured, as well as to discover other variables that might somehow predict or mediate this association between variables.

Following the analyses carried out, the researchers’ expectations were partially supported by the results, proving the existence of a significant relationship between perfectionism (socially prescribed perfectionism; perfectionistic self-promotion) and appearance-based rejection sensitivity and interest in undergoing an esthetic operation. There were no significant relationships between openness, emotional stability, extraversion, agreeableness, conscientiousness narcissism or self-oriented perfectionism and acceptance of cosmetic surgery. For Machiavellianism, psychopathy, introversion, neuroticism, non-display of imperfection, non-disclosure of imperfection, other oriented perfectionism, statistical analysis could not be performed because there were insufficient eligible studies for us to be able to study the relationship between them and the desire to undergo cosmetic surgery.

According to our meta-analysis, a person who wants to undergo cosmetic surgery may also have high levels of perfectionism (socially prescribed perfectionism and perfectionistic self-promotion; in some studies, a facet of perfectionism has been associated with neuroticism; Cruce et al., 2012; Newby et al., 2017). Such a person perceives the probability of being negatively evaluated by others as higher, relies heavily on what others think, and wants to be accepted by society (McCrae and Costa, 1987). Thus, given a high degree of neuroticism, it is possible that people with this profile want to align themselves with culturally imposed standards of beauty, to represent a model of perfection just so as to be accepted, a feature that leads to an increase in body dissatisfaction (Embacher Martin et al., 2018; Soohinda et al., 2019), a variable associated in the specialist literature with interest in esthetic operations (Walker et al., 2021; Kam et al., 2022). Along the same lines, people with a high level of appearance-based rejection sensitivity tend to be anxious because they believe they will be rejected due to their physical appearance (Park, 2007). Thus, with this personality trait, people may be more prone to internalize pressure and messages from parents, friends and the media regarding a handsome physical appearance (Webb and Zimmer-Gembeck, 2016; Webb et al., 2017) and, via a process of social comparison with the physique or face of other people regarded as beautiful (Rodgers et al., 2015; Shahyad et al., 2015), may end up developing a negative body image (Ridolfi et al., 2011; Warren and Rios, 2013) which, over time, can lead to the desire to undergo an esthetic operation to obtain a satisfactory physical appearance (Khabbaz Sabet et al., 2022; Nerini et al., 2022). More research will need to be carried out regarding the association between neuroticism and acceptance of cosmetic surgery, because it could represent an important factor for the decision to undergo cosmetic surgery (as we can see, neuroticism is associated with other traits which are related to acceptance of cosmetic surgery, but we could not find enough eligible studies to test this relationship).

No statistically significant relationship was observed between narcissism, extraversion, agreeableness, conscientiousness, openness, emotional stability or self-oriented perfectionism and interest in cosmetic surgery. In the case of narcissism, this may be due to the small number of studies on the subject. It is also possible that narcissism, as an isolated personality trait, is not sufficient to create a link with an acceptance of cosmetic surgery, and that it is for this reason that we find an association only between narcissistic personality disorder (the whole spectrum of narcissist specific traits) and attitudes toward cosmetic surgery (Loron et al., 2018; Jafferany et al., 2020). There was a positive but statistically insignificant relationship between agreeableness and interest in cosmetic surgery. However, Golshani et al. (2016) and Von Soest et al. (2006) maintain the existence of a relationship between agreeableness and acceptance of cosmetic surgery, on the grounds that people with a high level of this trait can be more easily influenced by those around them to take such a decision (Von Soest et al., 2009). According to our meta-analysis, the relationship between the two variables is not strong enough to produce a significant effect. Extraversion is in the same situation: the meta-analysis reveals a positive but insignificant link with interest in cosmetic surgery, so that no strong effect can be observed between the two. The specialist literature considers extraversion to be a determining factor for opting to have an esthetic operation (Javo and Sørlie, 2009), extraverts being regarded as agreeable people by their group of friends, so that they can discuss with and be influenced by friends in making such decisions (Öztürk, 2021). The link between conscientiousness and interest in cosmetic surgery is again positive, but insignificant, without producing any effect according to our study. Although this trait has not been extensively studied in association with interest in cosmetic surgery, self-realization as a facet of conscientiousness could explain a possible significant relationship with willingness to undergo cosmetic surgery (Genz, 2011; MacPherson, 2011). The association between openness and interest in cosmetic surgery is positive, but insignificant. People with high levels of openness to experience can have a much more favorable attitude toward these operations because he does not have too many mental barriers, and those that do exist are extremely flexible; they want to explore entirely new aspects and are also attracted toward the esthetic side (beauty, the arts) (McCrae and Greenberg, 2014). Having an inclination toward beauty and esthetics, but also being non-conformist, the person with a greater degree of openness to experience may feel the need to highlight certain parts of the body through esthetic operations precisely in order to create the impression of distinctiveness in relation to others (Tylka, 2018) or to increase self-confidence (Gillen and Dunaev, 2017). Also, the association between emotional stability (low neuroticism) and interest in cosmetic surgery is negative, but insignificant. Kvalem et al. (2006) mention that this personality trait cand be associated with the Intrapersonal factor from the ACSS scale, because those with high levels of neuroticism tend to experience negative affects which are associated with negative self-evaluations of appearance. The link between self-oriented perfectionism and interest to cosmetic surgery is positive but also insignificant, which can be due to the small numbers of studies on this subject. Sherry et al. (2005) characterized the self-oriented perfectionist as people who are very self-critical, aspect that can contribute to a high level of body dissatisfaction and generate interest in cosmetic surgery. More research will need to be carried out into this topic before a definite conclusion can be reached.

The results of the meta-analysis show the role that certain personality traits can play in a person’s decision to undergo cosmetic surgery. When someone possesses these traits, their predisposition to internalize messages from the three sources of influence regarding culturally imposed standards and to engage in self-objectification once the messages have been accepted can be much greater. As observed in the specialist literature, this process can lead to dissatisfaction related to body image (body dissatisfaction, shame), with the result that eating disorders and various body modification strategies may appear, one of which is resorting to esthetic operations. In addition to this, Davis (2003) points out that someone’s personality, emotions, and state of health can be observed from their physical appearance, with the signs of aging being associated – by those who stigmatize this natural process – with personality traits such as anger, fatigue, aggression and sadness. Thus, people who are often associated with such personality traits may be influenced by the opinion of those around them to undergo an esthetic operation to combat the marks of aging, a procedure that they hope will lead to their being perceived more positively (Honigman and Castle, 2006; Eriksen, 2012; Wu et al., 2022a,b). A qualitative study carried out on the Finnish population shows that people in that country who wish to undergo cosmetic surgery are motivated to change certain parts of the body so that they appear more extroverted and positive because their broad foreheads and drooping eyelids (two physical features that characterize Finns) give an impression of melancholy, introversion, and lack of agreeableness (Kinnunen, 2010). Also, Mohammadpanah et al. (2012) highlight the importance of psychological counseling before patients undergo cosmetic surgery, because this decision is closely related to defensive mechanisms and is based on a combination of psychological, emotional, and personal factors (those who wanted to undergo a cosmetic operation were immature and irrational when faced with difficulty and were less agreeable).

It is therefore vital to design interventions that highlight the risks that accompany the decision to undergo an esthetic operation. Borah et al. (1999) have identified a number of psychological problems (depression, anxiety, disappointment, sleep disturbances and post-traumatic stress syndrome) experienced by respondents after undergoing operations of this type. Brinton et al. (2006) also identified a higher risk of suicide among women who had had cosmetic surgery than among the general population. If we have an overview of how these variables relate (personality traits that predispose people to internalize messages from the media and to be interested in cosmetic surgery), interventions can be devised to make people critically analyze the information they receive or find on social networks and instruct them in how to distinguish between credible and less credible sources of information (fake news). The present meta-analysis shows the importance of personality structure for the taking of decisions about one’s own body. This relates to factors from the tripartite model of influence that predict interest in cosmetic surgery. There is therefore a need to study this theoretical framework in greater depth, looking at personality traits in an integrated way so as to better understand how this mechanism works, how we can assist those who are in distress due to the fact that they want to change their appearance, and how to help them identify the reasons why they no longer feel good about themselves.

## Limitations

While the studies investigated in the analysis partially confirmed the researchers’ expectations, the results must be interpreted with great caution, since the relatively small number of studies means that it is not possible to draw general conclusions from them; this represents a first limitation. This paucity of relevant studies may be because the field of cosmetic surgery in relation to personality traits in the non-clinical population is such a new one that few researchers have yet looked at the relationships between these variables. By contrast, the relationship between body image and interest in cosmetic surgery has been studied much more comprehensively.

Another limitation could be the fact that one of the eligibility criteria required studies to have been subjected to a peer-review process, so there is a possibility that relevant dissertations and other unpublished research work will have been omitted from the meta-analysis.

A further limitation could be the fact that not all the studies measured all the targeted constructs with the help of standardized instruments (there are relatively few instruments that measure esthetic surgery constructs that could be associated with variables of interest), which suggests there is a need to carry out work to design and validate such instruments in as many countries as possible.

A final limitation is the fact that not all the variables were operationalized in the same way. What is needed is a specific theoretical model for this topic that will include all the factors that can influence a person’s decision to undergo cosmetic surgery.

## Conclusion

Interest in cosmetic surgery has grown enormously in recent times. People want to feel good about their bodies and to achieve their ideal of beauty, but sometimes this desire is so strong that the risks that accompany making such a decision are no longer taken into account. Cosmetic surgeons should familiarize themselves with their patients’ personality traits and characteristics before modifying body parts, precisely in order to prevent any adverse results. Napoleon (1993) developed a guide in which he describes, in detail, both the behaviors that the esthetic surgeon and his team should adopt and also those that should be avoided when dealing with people with particular personality disorders, in order for the intervention to have the hoped-for effect. Thus, for example, those who are narcissistic need to be validated and praised by the cosmetic surgeon; the surgeon must appear self-confident, because the narcissist regards him as an extension of himself; it is desirable that the surgeon should not use the kind of everyday language that would be normal with other patients. Again, those who are obsessive-compulsive respond very well to precise, well-defined instructions, and it is desirable that the medical team should give them all the time they need, because otherwise their impatience to move on could be seen as a sign of rejection. Histrionics demand special attention, so a detached, analytical style on the part of the medical team will be counterproductive. Such a guidelines should be developed for those people who want to undergo cosmetic surgery and have different types of personality traits (for example, perfectionist self-promotion, socially prescribed perfectionism, appearance-based rejection sensitivity), but are not diagnosed with a specific disorder.

It goes without saying that some of the personality traits investigated in this meta-analysis may be associated with certain behaviors specific to personality disorders. It is important that esthetic surgeons and psychotherapists should know what approach to adopt with clients when working with them regarding their desire to undergo cosmetic surgery. However, the current situation is that cosmetic surgeons in this position have only personality *disorder* guidelines to rely on. The present meta-analysis highlights the need for psychologists and psychotherapists to develop guidelines giving productive behaviors and approaches for cosmetic surgeons to adopt when people from the non-clinical population who have different personality traits walk into their offices requesting esthetic surgery.

## Data availability statement

The original contributions presented in the study are included in the article/supplementary material, further inquiries can be directed to the corresponding author.

## Author contributions

MV and G-ML: conceptualization, methodology of the study, and data analysis. G-ML: data collection, data proofing, and manuscript writing. MV: manuscript review. All authors contributed to the article and approved the submitted version.

## Funding

Open access publication fees received from West University of Timisoara.

## Conflict of interest

The authors declare that the research was conducted in the absence of any commercial or financial relationships that could be construed as a potential conflict of interest.

## Publisher’s note

All claims expressed in this article are solely those of the authors and do not necessarily represent those of their affiliated organizations, or those of the publisher, the editors and the reviewers. Any product that may be evaluated in this article, or claim that may be made by its manufacturer, is not guaranteed or endorsed by the publisher.

## Appendix

*Search strategy and databases*

*Science Direct*: Advanced Search – Show all fields – Title, Abstract or Author-specified keyword – (“cosmetic surgery” OR “consideration of cosmetic surgery” OR “acceptance of cosmetic surgery” OR “interest in cosmetic surgery”) AND (personality OR “personality traits” OR “traits” OR “personality theory”).

*Scopus:* (“cosmetic surgery” OR “consideration of cosmetic surgery” OR “acceptance of cosmetic surgery” OR “interest in cosmetic surgery”) AND (personality OR “personality traits” OR “traits” OR “personality theory”) – Scopus database has by default the option to apply the search strategy to the abstract, title and keywords.

*Web of Science:* Topic Field (selecting this option we could apply to the abstract, title and keywords the search strategy) – (“cosmetic surgery” OR “consideration of cosmetic surgery” OR “acceptance of cosmetic surgery” OR “interest in cosmetic surgery”) AND (personality OR “personality traits” OR “traits” OR “personality theory”).

*Springer:* (“cosmetic surgery” OR “consideration of cosmetic surgery” OR “acceptance of cosmetic surgery” OR “interest in cosmetic surgery”) AND (personality OR “personality traits” OR “traits” OR “personality theory”) – Basic search has by default the option to apply the search strategy to the abstract, title and keywords.

*PubMed:* (“cosmetic surgery” OR “consideration of cosmetic surgery” OR “acceptance of cosmetic surgery” OR “interest in cosmetic surgery”) AND (personality OR “personality traits” OR “traits” OR “personality theory”) – Advanced Search to choose the needed fields.

*PsycInfo*: (“cosmetic surgery.tw” OR “consideration of cosmetic surgery.tw” OR “acceptance of cosmetic surgery.tw” OR “interest in cosmetic surgery.tw”) AND (personality.tw OR “personality traits.tw” OR “traits.tw” OR “personality theory.tw”) – the.tw function allows to apply to the abstract, title and keywords the search strategy.
